# Rainbow Smelt (*Osmerus mordax*) Genomic Library and EST Resources

**DOI:** 10.1007/s10126-008-9089-6

**Published:** 2008-04-02

**Authors:** K. R. von Schalburg, J. Leong, G. A. Cooper, A. Robb, M. R. Beetz-Sargent, R. Lieph, R. A. Holt, R. Moore, K. V. Ewart, W. R. Driedzic, B. F. H. ten Hallers, B. Zhu, P. J. de Jong, W. S. Davidson, B. F. Koop

**Affiliations:** 1 Centre for Biomedical Research, University of Victoria, Victoria, BC V8W 3N5 Canada; 2 Genome Sciences Centre, BC Cancer Agency, Vancouver, BC V5Z 4S6 Canada; 3 Institute for Marine Biosciences, National Research Council, Halifax, NS B3H 3Z1 Canada; 4 Oceans Sciences Centre, Memorial University of Newfoundland, St. John’s, NF A1C 5S7 Canada; 5 BACPAC Resources, Children’s Hospital Oakland, 747 52nd St., Oakland, CA 94609 USA; 6 Molecular Biology and Biochemistry, Simon Fraser University, Burnaby, BC V5A 1S6 Canada; 7 Department of Biology, University of Victoria, P.O. Box 3020, Victoria, BC V8W 3N5 Canada

**Keywords:** cDNA, EST database, BAC library, Rainbow smelt

## Abstract

Genomic resources in rainbow smelt (*Osmerus mordax*) enable us to examine the genome duplication process in salmonids and test hypotheses relating to the fate of duplicated genes. They further enable us to pursue physiological and ecological studies in smelt. A bacterial artificial chromosome library containing 52,410 clones with an average insert size of 146 kb was constructed. This library represents an 11-fold average coverage of the rainbow smelt (*O. mordax*) genome. In addition, several complementary deoxyribonucleic acid libraries were constructed, and 36,758 sequences were obtained and combined into 12,159 transcripts. Over half of these transcripts have been identified, several of which have been associated with cold adaptation. These basic resources show high levels of similarity (86%) to salmonid genes and provide initial support for genome duplication in the salmonid ancestor. They also facilitate identification of genes important to fish and direct us toward new technologies for other studies in fish biology.

## Introduction

Osmeriformes are close relatives of the Salmoniformes. The Osmeroidei and Salmoniformes clades are separated by at least 200 My with the Salmonidae having undergone at least one genome duplication event since their divergence (Ohno et al. 1968; Allendorf and Thorgaard 1984; Ishiguro et al. 2003). Osmerids, such as the rainbow smelt, have less than half the amount of genomic deoxyribonucleic acid (DNA) as salmonids and are considered to represent the ancestral state prior to the salmonid genome duplication (Ohno 1970). The bacterial artificial chromosome (BAC) resources provide a unique opportunity to study differences between orthologs (and paralog numbers), as well as chromosome alterations (through syntenic BAC comparisons), between species.

Rainbow smelt and salmon are closely related and have similar life histories; however, they represent different scales of cold adaptation. Smelt, unlike salmonids, are completely cold adapted, fully freeze resistant, active, and feed voraciously at low temperature (reviewed by Driedzic and Ewart 2004). Smelt have adapted to these conditions by producing and accumulating an antifreeze protein (AFP), glycerol, trimethylamine *N*-oxide, and urea that each contribute to lowering the freezing point of the fish (Driedzic and Ewart 2004). Glycerol can be synthesized from glucose or amino acid precursors in smelt (Walter et al. 2006). It is interesting to note that the abbreviated pathway by which glycerol is produced from amino acids is well known in mammals and termed glyceroneogenesis (Hanson and Reshef 2003). The seasonal accumulation of glycerol and AFP do not appear to be linked transcriptionally or metabolically (Liebscher et al. 2006).

To isolate and identify genes involved in cold adaptation and other physiological functions, we have constructed a large BAC clone and BAC library and generated a large expressed sequence tag (EST) clone and sequence database. Our large smelt EST resource facilitates further gene discovery and determination of how genes (proteins) evolve new functions and processes between species and provide an opportunity for future microarray and microsatellite studies.

## Materials and Methods

### BAC Resources

To provide a genomic clone resource, a BAC library, CHORI-74, was prepared following Osoegawa et al. (1998; Children’s Hospital Oakland Research Institute [CHORI], Oakland, CA, USA). High-molecular-weight DNA was isolated from blood cells from a female individual, ID number 4, partially digested with a combination of *Eco*RI restriction and *Eco*RI methylase enzymes and then size fractionated by pulsed-field gel electrophoresis. DNA fragments were cloned into the pBAC-GMR vector. The library was arrayed into 144 384-well microtiter plates and gridded onto three 22 × 22-cm nylon high-density filters. Each hybridization membrane represents more than 18,000 distinct BAC clones, stamped in duplicate.

### EST Resources

To identify genes in *Osmerus mordax*, complementary DNA (cDNA) libraries were constructed from ribonucleic acid (RNA) isolated from samples obtained from the Memorial University of Newfoundland Ocean Sciences Center, Logy Bay, NL, Canada. Smelt were collected in October 2002 in Long Harbour, Placentia Bay, Newfoundland, then transferred to the Ocean Sciences Centre, held under a natural photoperiod, and fed chopped herring twice per week. Fish were maintained in seawater at ambient temperature, which followed a profile similar to that presented in Lewis et al. (2004). Fish were sampled in January and April 2003. Brain, liver, head kidney, and spleen tissues were flash frozen and stored at −80°C until RNA extraction. Total RNA (Trizol reagent; Invitrogen, Carlsbad, CA, USA) or poly(A)+ RNA (FastTrack MAG kit; Invitrogen) was extracted from the flash-frozen tissues. Conventional libraries of low- and high-molecular-weight smelt brain, liver, kidney, and spleen cDNAs were individually constructed using pBluescript II XR cDNA library construction kits (Stratagene, La Jolla, CA, USA). Mixed tissue libraries were normalized by either the negative subtraction-based normalization method (Invitrogen; Research Genetics, California) or the duplex-specific nuclease normalization method (Evrogen, Moscow, Russia). The normalized libraries were directionally constructed in pCMV-Sport6.1 (Invitrogen) or pAL-17.3 (Evrogen) vectors.

### Bioinformatic Resources

Plasmid DNAs were extracted and BigDye™ Terminator (ABI, Foster City, CA, USA) cycle sequenced on ABI 3730 sequencers using conventional procedures and the following primers: 5′-T_18_-3′, M13 forward (5′-GTAAAACGACGGCCAGT-3′), and M13 reverse (5′-AACAGCTATGACCATG-3′ or 5′-CAGGAAACAGCTATGAC-3′). Base calling and trimming of vector, poly-A tails, and low-quality regions were addressed as described by Rise et al. (2004). Initial assembly of ESTs into contigs used PHRAP (http://bozeman.mbt.washington.edu), under stringent clustering parameters (minimum score = 100; repeat stringency = 0.99). A second-stage assembly used the consensus sequences (with quality scores) from the first stage and parameters of 96% repeat frequency and 300 minscore to build final contigs and consensus sequences. Assemblies using CAP3 (Huang and Madan 1999) using default parameters of 75% identity over an area of 30 bp resulted in similar contigs. Contig consensus sequences and singleton sequences were aligned with nonredundant GenBank nucleotide and several amino acid sequence databases (Gene Ontology [GO], swissprot, Conserved Domain Database [CDD], and Uniref90) using BLASTN and BLASTX, respectively (Boguski et al. 1993; Altschul et al. 1997; Schwede et al. 2003; Camon et al. 2004; Harris et al. 2004; Marchler-Bauer et al. 2005; Kopp and Schwede 2006). Using the swissprot database cross-reference, alignments of the second-stage contigs with entries in the database were used to assign GO terms to the contigs.

The EST resources have been submitted to GenBank with the following accession numbers: for the normalized libraries, EL518196 to EL551831, and for the non-normalized libraries, CB484654 to CB484815, CN442489 to CN442491, CX349771 to CX351193, and EL517809 to EL518195. Sequence databases, assemblies, consensus sequences, tools such as BLAST, and sequence and consensus annotations are available at the Genomics Research on Atlantic Salmon Project website (http://www.uvic.ca/cbr/grasp).

## Results and Discussion

**Fig. 1:**
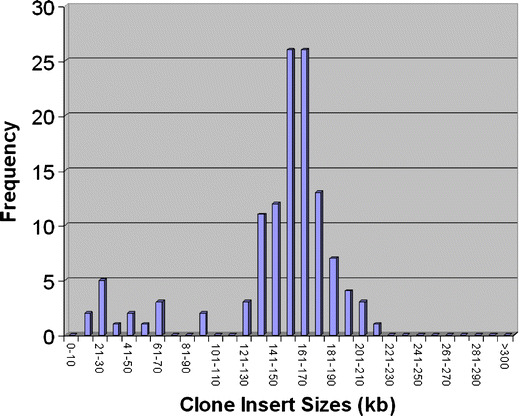
The insert size distribution

In the study, 52,410 BAC clones with an average insert size of 146 kb were obtained. Determination of the average insert size was calculated by taking one sample from each plate and, following minipreping and *Not*1 digestion, sizing by contour-clamped homogeneous electric field electrophoresis (CHORI). The insert size distribution is shown in Fig. 1. Given an estimated genome size of 0.69 pg (Hardie and Hebert 2003), the BAC clone library represents approximately 11-fold genome coverage. These BAC clones enable us to isolate and characterize gene regions of interest and are available through CHORI BAC resources (http://bacpac.chori.org/library.php?id=421).

At least 33,636 sequences were sequenced from the two normalized, mixed tissue libraries, and these were combined with 1,975 ESTs from the non-normalized libraries for a total of 35,611 EST sequences submitted to GenBank. Of the 12,159 second-stage contigs or transcripts assembled from 36,758 EST sequences, 6,139 had a BLASTX hit with *E* values less than 1e−10 to a well-annotated protein entry in the swissprot, CDD, or Uniref90 database (Table 1).

**Table 1:** Rainbow smelt EST project summary

	Rainbow smelt
Number of EST sequences^a^	36,758
Assembly stage 1^b^	
Number of contigs (2+ seq)^c^	9,044
Number of singletons^d^	7,019
Number of transcripts^e^	16,063
Max. contig size^f^	185
Ave. contig size^g^	2.29
Number of transcripts with BLASTX hits^h^	8,457
Assembly stage 2	
Number of transcripts^i^	12,159
Number with BLASTX hits^h^	6,139
Percent with hits^j^	50

^a^Number of EST sequences for all of the species including those in GenBank as of September 2007

^b^Assembly stage 1 refers to PHRAP assembly using parameters 99 repeat_frequency and 100 minscore

^c^Number of contigs with two or more sequences

^d^Number of contigs with one sequence

^e^Total number of transcripts including singletons

^f^The largest number of sequences that are contained within any single contig

^g^The average number of sequences within each contig (includes singletons)

^h^Number of transcripts that have a BLASTX hit of less than 1e−10 to swissprot databases

^i^The number of transcripts that result from a reassembly of all stage 1 transcripts using PHRAP parameters 96 repeat_frequency and 300 minscore

^j^Percent of stage 2 assembled transcripts that have a BLASTX hit

Alignments of the 6,139 contigs to entries in the swissprot database led to a total of 9,921 GO annotations to 2,500 different terms. The three ontologies comprising GO—molecular function, biological process, and cellular component—provided annotations for 3,534, 3,846, and 2,506 contigs, respectively. A further breakdown of the annotations is provided in Table 2. The complete GO hierarchy and the annotations corresponding to the contigs are available at http://www.uvic.ca/cbr/grasp.

**Table 2:** GO annotation of contigs

GO accession	GO term name	Number of contigs
Biological process ontology		
GO:0000003	Reproduction	88
GO:0002376	Immune system process	97
GO:0008152	Metabolic process	1,370
GO:0009987	Cellular process	2,869
GO:0016032	Viral reproduction	6
GO:0022414	Reproductive process	45
GO:0022610	Biological adhesion	85
GO:0032501	Multicellular organismal process	581
GO:0032502	Developmental process	671
GO:0040007	Growth	34
GO:0040011	Locomotion	15
GO:0043473	Pigmentation	5
GO:0048511	Rhythmic process	11
GO:0050896	Response to stimulus	373
GO:0051179	Localization	459
GO:0051234	Establishment of localization	373
GO:0051235	Maintenance of localization	8
GO:0051704	Multiorganism process	37
GO:0065007	Biological regulation	911
Cellular component ontology		
GO:0005576	Extracellular region	91
GO:0005623	Cell	2,401
GO:0031012	Extracellular matrix	25
GO:0031974	Membrane-enclosed lumen	162
GO:0031975	Envelope	105
GO:0032991	Macromolecular complex	337
GO:0043226	Organelle	1,272
GO:0044420	Extracellular matrix part	11
GO:0044421	Extracellular region part	66
GO:0044422	Organelle part	592
GO:0044456	Synapse part	6
GO:0045202	Synapse	11
Molecular function ontology		
GO:0003774	Motor activity	16
GO:0003824	Catalytic activity	1,078
GO:0005198	Structural molecule activity	53
GO:0005215	Transporter activity	140
GO:0005488	Binding	1,963
GO:0015457	Auxiliary transport protein activity	11
GO:0016209	Antioxidant activity	13
GO:0030188	Chaperone regulator activity	7
GO:0030234	Enzyme regulator activity	119
GO:0030528	Transcription regulator activity	210
GO:0031386	Protein tag	1
GO:0045182	Translation regulator activity	21
GO:0060089	Molecular transducer activity	140

For molecular function, 1,078, 1,635, and 497 contigs have been ascribed catalytic, nucleotide- and protein-binding, or regulator and transducer activities, respectively. The cellular component presents contigs that comprise various cellular regions, partitioning representatives to extra- or intracellular regions, as well as to mitochondrial, endoplasmic reticular, or nuclear regions. For the 3,846 contigs assigned a biological process, 1,370 represented metabolism of macromolecules, proteins, and lipids, and 2,869 represented cellular processes, such as reproductive, immune system, cell communication, cell cycle, proliferation, and development (including morphogenesis, differentiation and localization) processes (Table 2).

When the 12,159 contigs were compared (BLASTN) to EST sequences in GenBank, 4,697 rainbow smelt transcripts aligned with an Atlantic salmon EST (*E* value less than 1e−25 over more than 200 bp) with an average identity of 86.2% (over an average of 431 bp), and 4,347 transcripts aligned with rainbow trout ESTs with an average identity of 86.1% (over 419 bp). These comparisons provide only a very general indication of the similarity between transcriptomes of rainbow smelt and salmonids, as assemblies contain both 5′- (generally genic regions) and 3′- (generally 3′-untranslated regions) transcript reads. However, these DNA sequence similarity values corroborate a more ancient separation of rainbow smelt and salmonid species than duplicated salmonid major histocompatibility complex class IA and B genes (Lukacs et al. 2007) or growth hormone genes (McKay et al. 2004). Comparisons of sequence identity between the Atlantic salmon gene duplicates are closer to one another (88% to 95%) than to any of the aligned smelt EST sequences (86%), consistent with an ancestral salmonid genome duplication hypothesis. Moreover, the high level of similarity between rainbow smelt ESTs and salmonid ESTs (86% identity) explains the observed high level of rainbow smelt cDNA hybridization to salmonid cDNA microarrays (Rise et al. 2004; von Schalburg et al. 2005).

The primary function of the AFP in smelt tissues is likely to be freezing point depression, although roles for AFPs in low-temperature tolerance have also been suggested (reviewed by Inglis et al. 2006). Seasonal expression of smelt AFP has been shown (Liebscher et al. 2006). However, the tissue distribution of expression was unknown. Our liver libraries predominately contained type II AFP transcripts. In fact, sequences representing AFP clustered to one contig with the highest frequency of all genes in the smelt database. The AFP does not appear to be expressed in the brain, head kidney, or spleen libraries, suggesting that the liver is exclusive or predominant among these tissues in expressing AFP in smelt. Further insight into the evolution, diversity, and structure/function of the smelt AFP may arise from studies using the resources developed here.

Cold adaptation is normally multifactorial, and it is likely that smelt have adaptations in addition to the known glycerol and AFP. Studies to identify other adaptations will draw largely on the resources presented here. The opportunity to further study low-temperature adaptation in this thoroughly cold adapted vertebrate may present unique opportunities for new applications in animal biology and in medicine.
